# Supplementary material for: EAD: effortless anomalies detection, a deep learning based approach for detecting outliers in English textual data
Source: PeerJ Comput Sci. 2024 Nov 13;10:e2479. doi: 10.7717/peerj-cs.2479 (PMC11623099; doi:10.7717/peerj-cs.2479)
Supplement: Supplemental Information 2 [file peerj-cs-10-2479-s002.docx]

**Motivation:**

The motivation for this research is to address the inefficiencies of current anomaly detection methods by proposing an innovative, unsupervised approach that leverages advanced natural language processing techniques. Specifically, the study introduces a MiniLM-based system for outlier detection that employs centroid embeddings to extract anomalies from extensive datasets efficiently. This approach aims to reduce computational costs while maintaining high accuracy in detecting outliers.

**Reproducibilityity**

**Computing Infrastructure**

1. **Operating System:**
   - **Platform:** The study was conducted on a system running Ubuntu 20.04 LTS. Ubuntu was chosen for its stability, wide usage in the research community, and robust support for Python-based machine learning libraries.
2. **Hardware:**
   - **Processor (CPU):** Intel Core i7-10700K 3.8 GHz. This high-performance processor is capable of handling the computational demands of anomaly detection algorithms, especially when processing large datasets.
   - **Graphics Card (GPU):** NVIDIA GeForce RTX 3060. The GPU accelerates the training and inference processes of machine learning models, significantly reducing computation time.
   - **Memory (RAM):** 32 GB DDR4. Ample memory is essential for handling large datasets and complex model computations without running into memory bottlenecks.
   - **Storage:** 1 TB NVMe SSD. A solid-state drive ensures fast data access speeds, which is critical for large-scale data processing and efficient model training.

**Software**

1. **Programming Language:**
   - **Python 3.8:** Python was used due to its extensive library support for machine learning and data processing tasks, along with its ease of use and readability.

**Dataset (DOI/URL)**

Provided in the supplementary file.

**Description and Justification for model used**

**Description of the Model**

1. **MiniLM for Tokenization and Embedding:**
   - **MiniLM (Miniature Language Model):** MiniLM is a pre-trained, transformer-based language model that provides efficient and high-quality text embeddings. It is designed to deliver competitive performance with significantly fewer parameters compared to larger models like BERT or GPT.
   - **Tokenization:** MiniLM tokenizes text data into smaller units (tokens) and transforms these tokens into dense vector representations (embeddings) that capture semantic meaning.
   - **Centroid Embeddings:** These embeddings are used to compute centroid representations for clusters of data, which serve as reference points for detecting anomalies.

**Justification for the Model**

1. **Efficiency and Performance:**
   - **MiniLM’s Efficiency:** MiniLM provides a good balance between model size and performance. It offers fast processing and requires less computational power compared to larger models, making it well-suited for handling large datasets and real-time applications.
   - **Scalability:** The reduced computational cost of MiniLM allows the model to scale effectively with increasing data volume and variety, which is critical for anomaly detection in large datasets.

**Assessment metrics (justification)**

To evaluate the performance of the proposed unsupervised MiniLM-based anomaly detection system, several key metrics are used. These metrics provide a comprehensive view of the model’s effectiveness, accuracy, and robustness in identifying anomalies within large and diverse datasets.

**1. Accuracy**

- **Justification:** Accuracy is a fundamental metric for any classification task, including anomaly detection. It provides a clear indication of the model’s overall ability to correctly classify data points as either normal or anomalous. However, in the context of anomaly detection, accuracy alone might be insufficient due to class imbalance, as anomalies are typically much rarer than normal instances.

**2. Precision**

- **Justification:** Precision is critical in anomaly detection as it indicates the proportion of identified anomalies that are actual anomalies. High precision means fewer false positives, which is important in applications like fraud detection where false alarms can be costly and disruptive.

**F1 Score**

- **Justification:** The F1 score is particularly useful in scenarios with class imbalance, as it takes into account both false positives and false negatives. It provides a single metric that balances precision and recall, offering a more comprehensive evaluation of the model’s performance in detecting anomalies.
